# A Fast Image Deformity Correction Algorithm for Underwater Turbulent Image Distortion

**DOI:** 10.3390/s19183818

**Published:** 2019-09-04

**Authors:** Min Zhang, Yuzhang Chen, Yongcai Pan, Zhangfan Zeng

**Affiliations:** School of Computer Science and Information Engineering, Hubei University, Wuhan 430062, China (M.Z.) (Y.P.) (Z.Z.)

**Keywords:** underwater turbulence, image distortion, pixel shift, kernel correlation filtering algorithm

## Abstract

An algorithm correcting distortion based on estimating the pixel shift is proposed for the degradation caused by underwater turbulence. The distorted image is restored and reconstructed by reference frame selection and two–dimensional pixel registration. A support vector machine-based kernel correlation filtering algorithm is proposed and applied to improve the speed and efficiency of the correction algorithm. In order to validate the algorithm, laboratory experiments on a controlled simulation system of turbulent water and field experiments in rivers and oceans are carried out, and the experimental results are compared with traditional, theoretical model-based and particle image velocimetry-based restoration and reconstruction algorithms. Using subjective visual evaluation, image distortion has been effectively suppressed; based on an objective performance statistical analysis, the measured values are better than the traditional and formerly studied restoration and reconstruction algorithms. The method proposed in this paper is also much faster than the other algorithms. It can be concluded that the proposed algorithm can effectively improve the de-distortion effect of the underwater turbulence degraded image, and provide potential techniques for the accurate operation of underwater target detection in real time.

## 1. Introduction

With the development of underwater imaging technology, underwater target recognition has been widely used in topographic survey and geomorphological observation [1]. In natural static water, the scattering and absorption characteristics of suspended particles are the main factors causing the degradation of an underwater image [2], limiting the underwater visible range to only a few tens of meters. Former studies focused on the degradation model, solving beam transmission and scattering problems to improve the image quality and distance [3,4]. However, in real water environments like rivers and oceans, the underwater visible distance decreases severely due to turbulent effects; the nonuniform variation of light field distribution results in image distortion [5,6], which makes turbulence the most important degradation factor in natural water imaging. Therefore, it is necessary to study the underwater turbulent degradation in depth.

Some scholars have studied the degradation of underwater turbulence and its image recovery processing. Hou et al. [7,8,9,10,11] used the underwater imaging degradation model to analyze the effects of suspended particles, turbulence, and path scattering on underwater optical imaging. Gero et al. [12,13,14] established laboratory and field underwater turbulence experimental systems, conducted on-site measurements, and analyzed the influence of optical turbulence on the resolution of underwater imaging systems with quantitative data. Matt et al. [15,16] established a turbulent environment experimental platform with changeability and reproducibility. The Doppler velocimeter and the particle image velocimetry (PIV) system were used to analyze the fluid field and the computational fluid dynamics model was used to compensate for the measurement results. Farwell et al. [17,18] studied the intensity and coherence distribution of turbulence on underwater beam propagation based on the ocean turbulence power spectrum model, and performed a large number of numerical calculations. 

There are three main types of research: theoretical calculation from the turbulent structure function and scattering characteristics; the establishment of an experimental system for simulating turbulence for laboratory and field measurement and analysis; and simulation experiments using the PIV method. Chen et al. have studied these three methods [19,20], and the results show that the turbulent flow field causes modulation transfer function (MTF) declines of the whole spatial frequency, and the path radiation and fluid media lead to a decrease of modulation contrast of the high spatial frequency. In fact, turbulence will affect imaging at both high and low frequencies due to the nonuniformity of light field, which causes image distortion. Therefore, it is necessary to study image processing methods that are specifically aimed at image distortion.

Hu et al. [21] proposed a method based on the motion field kernel regression. Holohan et al. [22] proposed the use of adaptive optics (AO) technology for image processing. Wen et al. [23] proposed an underwater image reconstruction method based on motion compensation for high-quality image block selection and denoising. Kumar et al. [24] proposed a two–stage image reconstruction method. In the first stage, the blind image quality (BIQ) metric and K–means clustering algorithm are used to select the reference frame and clear frame sequence, respectively. In the second stage, the pixel registration technology and two-dimensional interpolation technology are used to reconstruct the distorted image. Although this method can effectively alleviate the impact of turbulence, the computational complexity is high.

In terms of image distortion elimination, Ahn et al. [25] introduced a convolutional neural network into image distortion classification. Mao [26] proposed a 2D interpolation-based distortion correction technique for bistatic Synthetic Aperture Radar (SAR) polar format image formation. Sun et al. [27] proposed an improved cubic chirplet decomposition method based on linked scatterers to solve the distortion problem for shipborne bistatic ISAR. There are other studies on the elimination of image distortion in different fields [28,29]. From the above, it can be seen that the elimination of image distortion is generally to carry out reverse operations based on the cause of distortion, among which are popular methods such as the use of neural networks to identify the types of distortion.

Therefore, in this paper, a self-defined metric is used to select the reference frame and the input frame sequence of the short exposure image with high clarity. Pixel registration and two-dimensional registration algorithms are used to suppress the distortion. The kernel correlation filtering algorithm is used to improve the speed and efficiency of the algorithm, which can reduce the amount of calculation and improve the deformity removal effect at the same time.

## 2. Theory and Methods

### 2.1. Two-Dimensional Pixel Registration Algorithm

The reference frame and the input frame sequence are selected according to the sharpness value of captured image frames. The sharpness of image can be calculated by [24]:

(1) $$B={\left(\frac{\sum_{i=1}^{I}{\left(\sigma_{\eta }-\bar{P}\left(\eta ,\phi_{i}\right)\right)}^{2}}{I}\right)}^{\frac{1}{2}},$$

where $\sigma_{\eta }$ is the mean value of $\bar{P}\left(\eta ,\phi_{i}\right)$, I represents the number of directions selected, and $\bar{P}\left(\eta ,\phi_{i}\right)$ denotes the expected entropy of the image:

(2) $$\bar{P}\left(\eta ,\phi_{i}\right)=\frac{\sum_{r}P\left(r,\phi_{i}\right)}{S},$$

(3) $$P\left(r\right)=-\frac{\log_{2}\left(\sum_{m=1}^{R}{\overset{⌣}{D}}_{r}^{3}\left(m\right)\right)}{2},$$

where η∈[1,2,…,S] represents the size of the image, $\phi_{i}\in \left[\phi_{1},\phi_{2},\ldots ,\phi_{I}\right]$ is the measurement direction, r and m represent the discrete variables of time and frequency, respectively, R is the number of pixels, and $\overset{⌣}{D}\left(m\right)=D\left(m\right)\cdot D^{*}\left(m\right)$ represents the complex conjugate of D(m).

We define D as the wave structure function of turbulence [30]:

(4) $$D\left(\rho ,Z\right)=3.603\times 10^{-7}k^{2}z\varepsilon^{-1/3}\left(\chi_{T}/\omega^{2}\right)\rho^{5/3}\left(0.419\omega^{2}-0.838\omega +0.419\right),$$

where k=2π/λ is the wavenumber equation, λ is the wavelength (530 nm when calculated in this paper), and *ρ* is the distance between two points on the cross section perpendicular to the transmission direction.

The reference frame can be selected as the input frame with the highest sharpness value, and frames with higher sharpness are kept as the input frame sequence for subsequent image processing.

The pixel shifting of the input frame sequence relative to the reference frame is calculated using the backward mapping method:

(5) $$\begin{array}{l} R_{a}\left(a,b\right)=\left({\sum_{g=1}^{G}Q}_{a}\left(a,b,g\right)\right)/G\\ R_{b}\left(a,b\right)=\left({\sum_{g=1}^{G}Q}_{b}\left(a,b,g\right)\right)/G \end{array},$$

where $R_{a}$ and $R_{b}$ represent the mean values of pixel shift in the horizontal and vertical directions, respectively, g denotes a frame index, and G denotes the total number of reserved frame sequences.

The corrected shift of each pixel in all reserved input frames is derived from:

(6) $$\begin{array}{c} Q_{a}^{*}\left(a,b,g\right)=Q_{a}\left(a+R_{a}^{-1}\left(a,b\right),b+R_{b}^{-1}\left(a,b\right),g\right)+R_{a}^{-1}\left(a,b\right)\\ Q_{b}^{*}\left(a,b,g\right)=Q_{b}\left(a+R_{a}^{-1}\left(a,b\right),b+R_{b}^{-1}\left(a,b\right),g\right)+R_{b}^{-1}\left(a,b\right) \end{array},$$

where $Q_{a}^{*}$ and $Q_{b}^{*}$ are the corrected displacements in the horizontal and vertical directions. $R_{a}^{-1}$ and $R_{b}^{-1}$ represent the inverse of $R_{a}$ and $R_{b}$, respectively.

Then the corrected frames can be restored and reconstructed by:

(7) $$\begin{array}{l} f_{1}\left(a,b\right)=f_{g}\left(a+Q_{a}^{*},b+Q_{b}^{*}\right)\\ \overset{}{f_{gn+1}}=f_{g}*\int_{-\infty }^{\infty }\int_{-\infty }^{\infty }h^{-1}(a,b)e^{j2\pi }dadb \end{array},$$

(8) $$\begin{array}{l} f_{2}\left(a,b\right)=f_{g}\left(a\cos\theta -b\sin\theta +Q_{a}^{*},b\cos\theta +a\sin\theta +Q_{b}^{*}\right)\\ f_{{}_{gn+1}}=P[f_{gn}+\sum_{i=1}^{P}\lambda P(g_{i}-hf_{i})] \end{array},$$

where $f_{1}\left(a,b\right)$ represents the restored image, $f_{2}\left(a,b\right)$ represents the reconstructed image, $f_{g}$ represents the sequence of reserved frames, θ represents the angle of rotation, and h denotes the Gaussian estimation.

The recovered image can be used as the reference image for the next iteration. Through multiple iterations, the de-distortion effect will be better removed.

### 2.2. Support Vector Machine-Based Kernel Correlation Filtering Algorithm

When it comes to finding the optimal solution for Equations (7) and (8), the regularization constraint process can be used for limiting the iteration process, the main idea of which is to solve the mathematical ill-conditioned problem of finding the minimum value. The constraint algorithm in this paper combined the idea of kernel correlation filters (KCF) algorithm.

The expression of the regularization is as shown [31]:

(9) $$f(x)=\underset{n}{\min}{\sum_{i=1}^{M}\left(x_{i}-\left(n_{i}^{T}x+z\right)\right)}^{2}+x{\left\|nw\right\|}^{2},$$

where x, $x_{i}$ represent the original image and the observed image, $\underset{n}{\min}$ represents the minimum value, ξ represents the regularity factor, and ${\left\|nw\right\|}^{2}$ represents the penalty factor.

As a result, in the process of solving Equations (7) and (8), the goal of Equation (9) is to solve a best approximation solution of $f\left(x\right)=n^{T}x+z$ that can be defined as the Interval of functions. 

The distance between a point in the sample space and the classification hyper plane is calculated as follows:

(10) $$r=\frac{\left|n^{T}x+z\right|}{\left\|n\right\|},$$

The distance between the support vector and the hyper plane is called the “interval” of the support vector machine (SVM), which can be expressed as follows:

(11) $$r=\frac{2}{\left\|n\right\|},$$

The principle of a support vector machine is to maximize the interval, i.e., to minimize the $\frac{1}{2}{\left\|n\right\|}^{2}$. The constrained optimization problem of linear classification can be expressed as follows:

(12) $$\begin{array}{l} \min_{n,z}\frac{1}{2}{\left\|n\right\|}^{2}\\ s.t.y_{i}\left(n^{T}x_{i}+z\right)\geq 1,i=1,\ldots ,m \end{array},$$

Lagrangian functions can be constructed by introducing Lagrange multipliers into constraints:

(13) $$\begin{array}{l} \alpha_{i}\geq 0,i=1,2,\cdots ,N\\ L\left(n,z,\alpha \right)=\frac{1}{2}{\left|n\right|}^{2}-\sum_{i=1}^{N}\alpha_{i}\left[y_{i}\left(n\cdot x_{i}+z\right)-1\right] \end{array},$$

Then the extremum can be obtained by summing partial derivatives:

(14) $$\begin{array}{l} \frac{\partial L}{\partial n}=0\Rightarrow n=\sum_{i=1}^{n}\alpha_{i}y_{i}x_{i}\\ \frac{\partial L}{\partial z}=0\Rightarrow \sum_{i=1}^{n}\alpha_{i}y_{i}=0 \end{array},$$

α can be obtained by substituting the above two conditions into the formula:

(15) $$\begin{array}{l} L{\left(n,z,a\right)}_{\max}=\sum_{i=1}^{n}a_{i}-\frac{1}{2}\sum_{i,j=1}^{n}a_{i}a_{j}y_{i}y_{j}x_{i}{}^{T}x_{j}\\ s.t.\sum_{i=1}^{n}a_{i}y_{i}=0,i=1,2,\ldots ,n \end{array},$$

In turn, n can be solved as follows:

(16) $$n=\sum_{i=1}^{n}\alpha_{i}^{*}y_{i}x_{i},$$

In the design process of the algorithm, in order to use the fuzzy sample image to train the least squares classifier and simplify the computation, a circular matrix can be constructed. We set:

(17) $$\sum_{i=1}^{n}\alpha_{i}^{}={\left(XX^{T}+\beta I\right)}^{-1},$$

where β is the parameter that controls overfitting.

Assuming that H(x) is a i×i matrix, it can be obtained by cyclic shifting of a vector of I∗i, from which X can be obtained:

(18) $$X=H(x)=\left(\begin{array}{cccc} x_{0} & x_{2} & \cdots & x_{i}\\ x_{i} & x_{1} & \cdots & x_{i-1}\\ \vdots & \vdots & \ddots & \vdots \\ x_{2} & x_{3} & \cdots & x_{1} \end{array}\right),$$

The above matrix can be converted by $I=E^{K}E$, E is the constructed core function:

(19) $$X^{K}X=Ediag(x)diag(x^{*})E^{K},$$

Since the matrix is diagonal, Equation (11) can be converted to:

(20) $$X^{K}X=Ediag(x^{*}\cdot x)E^{K},$$

Then the discrete Fourier form of n can be obtained by substituting Equation (20) into Equation (16), which can greatly reduce the amount of computation in the training process of the least squares classifier.

In summary, the kernel correlation matrix constructed in Equation (18) is substituted into Equation (16) to speed up the constrained optimization of classification in support vector machine for calculating the best approximation solution to Equation (9). Therefore, the factors affecting the speed of the algorithm are determined by the constructed core matrix. The constructed core functions of the matrix include radial basis function, point product kernel, weighted core, and so on. In this paper, the radial basis function core is selected. However, the factors affecting the accuracy of the algorithm return to the solution of Equations (7) and (8), where B in Equation (1) determines the input of the algorithm, and the kernels of restoration and reconstruction algorithms also have an impact on the accuracy. Thus, when the velocity of turbulence or visibility changes, the initial estimation function in Equations (7) and (8) will be changed to improve the algorithm.

## 3. Experimental Results and Analysis

In order to further verify the effectiveness of the proposed method, the experimental data for this paper were obtained through the laboratory simulation of a turbulent environment and field tests in a real turbulent ocean environment.

Due to the relationship between image distortion and the turbulent velocity field, the image restoration method based on PIV velocity field measurement is used as a verification method in the laboratory experiment system. Considering the follow abilities and light scattering characteristics of the tracer particles, common particle bubbles (which also have the advantage of being nonpolluting) are selected as tracer particles to measure the flow velocity field distribution of underwater turbulence. The probability density function of the bubble motion displacement can be described by the probability density function of time:

(21) $$f_{s}(s)=f_{t}(t)\times (\frac{1}{\left|s^{\prime }(t_{1})\right|}+\frac{1}{\left|s^{\prime }(t_{2})\right|}+\cdots +\frac{1}{\left|s^{\prime }(t_{n})\right|})\;=\frac{1}{t}\times \frac{1}{\left|v\right|},$$

where $f_{t}(t)$ is the probability density function of time, which is a random variable subject to uniform distribution; $s^{\prime }(t)$ is the reciprocal of the relative displacement s(t); |v| is the speed of bubble motion, which can be estimated by the bubble dynamics equation; and t is the exposure time of the image sensor. Then the motion modulation transfer function of the bubble can be calculated by the one-dimensional Fourier transform of the probability density function of the relative displacement:

(22) $$MTF_{motion}(f)=\frac{1}{d}\int_{0}^{d}\exp(-i2\pi fs)ds=\sin c\left(\pi fd\right),$$

Thus, the MTF can be used as a priori knowledge of image restoration reconstruction algorithm.

In order to objectively analyze the processing results, this paper selects objective evaluation criteria of the non-reference ideal image as the quality assessment of image restoration and reconstruction, including the information capacity (IC), blur metric (BM), and gray average gradient (GMG). IC characterizes the richness of useful image information; the BM describes the degree of image distortion; the GMG reflects the image edge information. The larger the values of IC and GMG, the smaller the BM value, which denotes the better effects of image restoration and reconstruction. These evaluation criteria have been described in detail in previous articles published by the research team [20], and so will not be repeated here [19].

The BM is defined as follows:

(23) $$\begin{array}{l} BM=\max\left(sD_{vertical,}sD_{horizontal}\right),sD_{vertical}=\sum_{i,j=1}^{m-1.n-1}D_{vertical}\left(i,j\right),\\ sD_{horizontal}=\sum_{i,j=1}^{m-1,n-1}D_{horizontal}\left(i,j\right),\\ i\in \left(0,m-1\right),j\in \left(0,n-1\right),\\ \left\{ \begin{array}{l} D_{vertical}=\left|F\left(i,j\right)-F\left(i-1,j\right)\right|\\ D_{horizontal}=\left|F\left(i,j\right)-F\left(i,j-1\right)\right| \end{array}\right. \end{array},$$

where $D_{vertical}$ and $D_{horizontal}$ represent different images in the vertical and horizontal directions. F(i,j) is the pixel of coordinate (i,j) on the image plane, and (m,n) is the size of the image. Then the blur metric can be normalized by the range 0 to 1.

The IC is defined as follows:

(24) $$IC=\log_{2}\left\{1+\sum \frac{\log\left[p\left(i,j,d,\theta \right)\right]}{\log\left[\max\left(p\left(i,j,d,\theta \right)\right)\right]}\right\},$$

where p(i,j,d,θ) represents the correlation between pixels, i and j represent the coordinates of the pixel, d is the imaging distance, and θ represents the direction of association between the pixels.

The GMG is defined as follows:

(25) $$GMG=\frac{1}{\left(M-1)(N-1\right)}\sum_{i=1}^{M-1}\sum_{j=1}^{N-1}\sqrt{\frac{{\left[f(x,y+1)-f(x,y)\right]}^{2}+{\left[f(x+1,y)-f(x,y)\right]}^{2}}{2}},$$

where f(x,y) denotes the point at coordinate (x,y) on image plane, and (M,N) is the size of the image.

### 3.1. Laboratory Experiments

**Figure 1:**
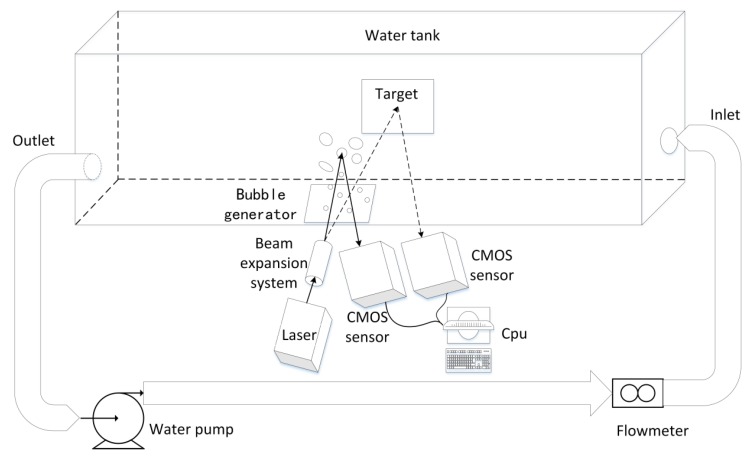
Stereoscopic structure diagram of laboratory experimental system.

An underwater turbulence experiment system is established in this paper. A 532 nm green semiconductor laser is used as the light source; images are captured by a high-speed COMS image sensor. The spot size of the laser is 10–20 mm, and its power is 200 mw. The experimental water tank is made of high-transmittance acrylic plate, so more than 90% of the laser source is irradiated on the target plate, and its size is 150 cm × 34 cm × 33 cm (length, height, width). Both the inlet and outlet are 40 mm round holes, at different heights to form turbulence with a water pump. The experimental system uses a circulating pump with a maximum head of 5 m and a maximum flow of 7.8 m^3^/h to provide hydrodynamic power. The laser and sensor are 33 cm away from the target plate. In order to reduce the experimental error, the experiment was carried out in a dark environment. The three-dimensional structure of the experimental system is shown in Figure 1.

The Reynolds number (Re) is used to determine whether the fluid is in a turbulent state. If Re >4000, the fluid state is turbulent. The flow rate of the water body is controlled by the flow meter and the water pump water valve. The pump drives the flow of water, and the valve controls the size of the flow. Turbulence occurs when the inlet flow reaches a certain speed. By controlling the water flow velocity at the water inlet of the water tank, turbulence of different strengths is obtained. The flow meter can read the velocity in real time, and then calculate the turbulent Reynolds number and turbulent intensity to ensure that the sample image is obtained in a turbulent environment.

The training platform of this algorithm is: the operating system is Ubuntu 14.04 (Canonical Ltd, London, England), the CPU is Core i7–9700K (Quad–core 4.9 GHz) (2200 Mission College Blvd. Santa Clara, CA 95054–1549 USA), and the graphics card is ASUS DUAL RTX2070–O8G–EVO (ASUS, Taipei City, Taiwan). The programming is performed in MATLAB R20017b (Apple Hill Drive, Natick, MA 01760–2098, USA). If the computer configuration is reduced or improved, the algorithm time will increase or decrease accordingly. If the image resolution increases, the number of training window travels in SVM will increase, and the algorithm time will increase accordingly. The image resolution of the sample images selected in this paper is cut to 800 × 600, and the scale factor of super-resolution reconstruction is set to 3.

#### 3.1.1. Microturbulent Environment

**Figure 2:**
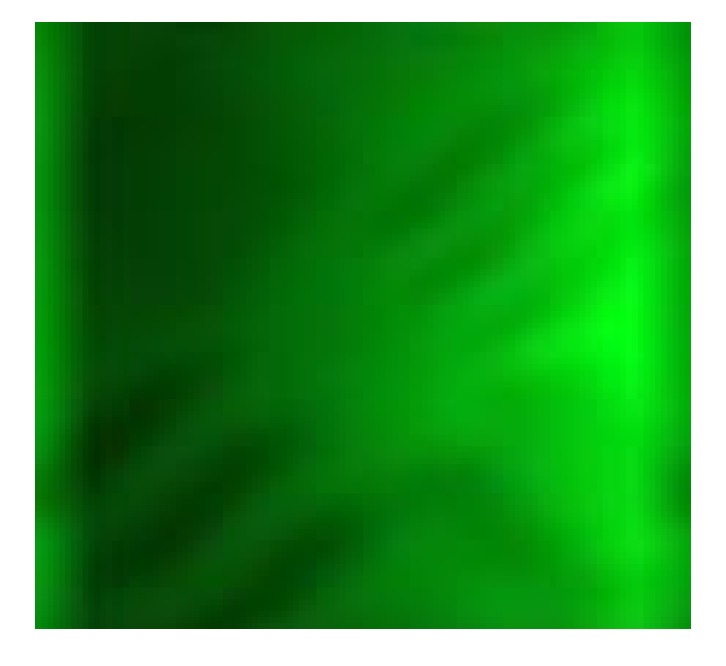
Sample image.

**Figure 3:**
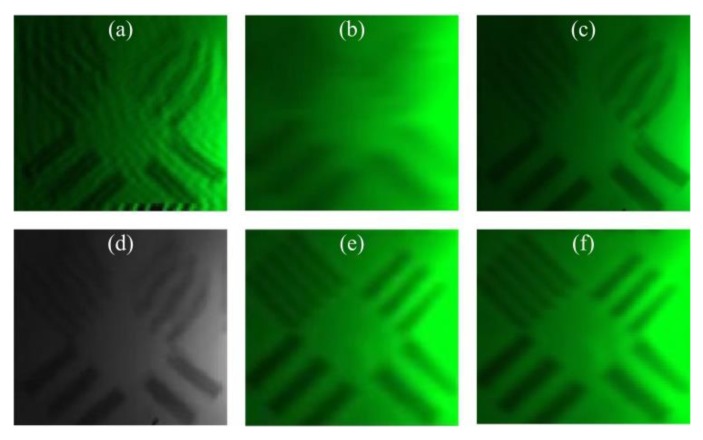
Restoration and reconstruction results: (**a**) result of BD; (**b**) result of POCS; (**c**) result of M−SB; (**d**) result of M−TV; (**e**) result of PIV−RR; (**f**) result of the proposed method in this paper.

When the water velocity of the inlet is 5 m/s, the target object is photographed 60 times by Charge Couple Device (CCD) sensor in 5 s. The captured image sequences are processed and compared by the proposed algorithm along with traditional blind restoration (BD) [32], projection onto convex set reconstruction (POCS) [33], the semi-blind restoration and reconstruction method based on the turbulent degradation model (M−SB) [19], the total variation image super-resolution reconstruction technique based on L1 norm (M−TV) [34], and a restoration and reconstruction method based on the PIV method (PIV−RR) [35]. The sample image taken is shown in Figure 2, with restored and reconstructed results shown in Figure 3. The evaluation values for the images are listed and compared in Table 1. Table 2 shows the processing time of the algorithms.

It can be seen that the traditional BD method relieved a certain degree of blurring and introduced a large ringing effect, which is improved by the M−SB method, but the distortion of the image is not improved. The POCS and M−TV methods can improve the image resolution while improving the image sharpness, but the distortion of the image is also not improved. It can be seen that the image restoration and reconstruction algorithms have a significant effect in terms of improving resolution and deblurring, but they are not suitable for image distortion. This explains the necessity of the algorithm that is proposed by this paper. Based on the method in this paper, the distortion can be significantly improved, and the PIV−RR method also has a certain effect on the processing of image distortion. 

As can be seen from Table 1, the BM values of M–SB and PIV–RR method are smaller compared to those obtained by the other methods. Although the BM value of the method proposed in this paper is larger than that of the other two methods, it is not much larger. The IC value of the M−TV method is the largest, which is followed by the proposed method, while the PIV−RR method has a small value. The GMG value of both PIV−RR and the proposed method are larger than those of the other methods. It can be concluded that the proposed algorithm is not as good at deblurring as targeted image restoration, but it has advantages over the PIV−RR method in reconstruction.

As can be seen from Table 2, the method proposed in this paper has obvious advantages in terms of the processing time. 

#### 3.1.2. Strong Turbulence Environment

**Figure 4:**
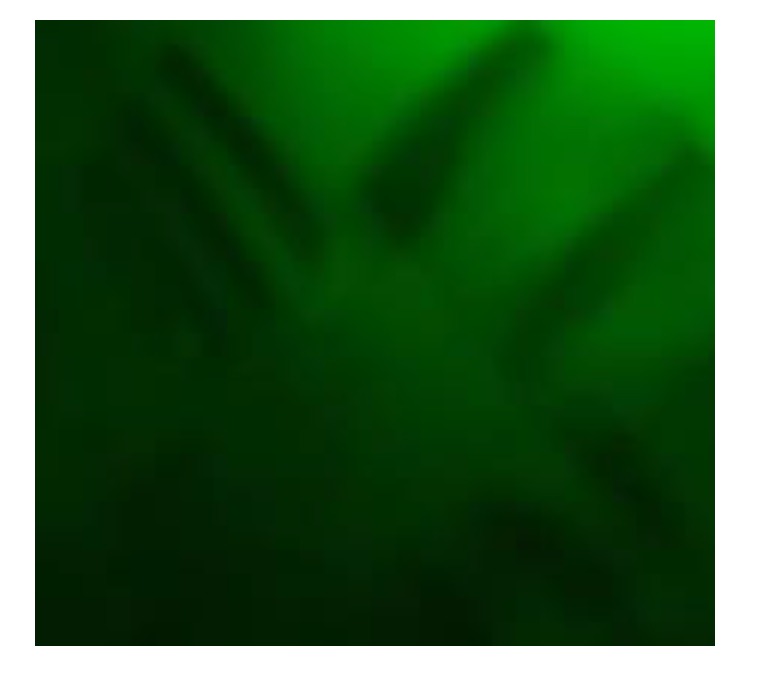
Sample image.

**Figure 5:**
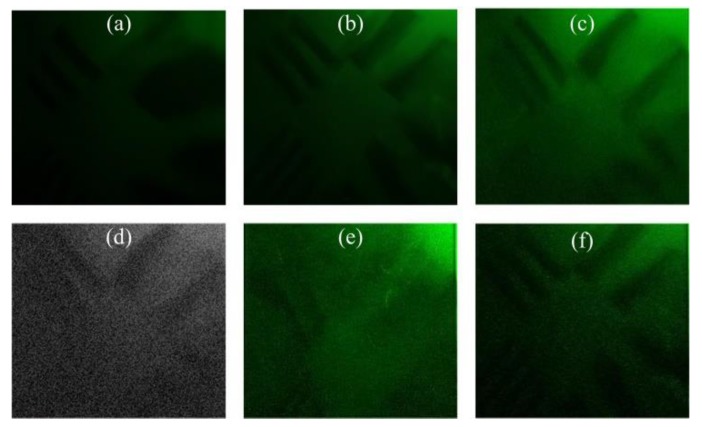
Restoration and reconstruction results: (**a**) result of BD; (**b**) result of POCS; (**c**) result of M−SB; (**d**) result of M−TV; (**e**) result of PIV−RR; (**f**) result of the proposed method in this paper.

When the water velocity of the inlet reaches 25 m/s, as can be seen in Figure 4, the degree of distortion of the image is greatly increased. The results after image restoration and reconstruction are shown in Figure 5. The evaluation values for the images are listed and compared in Table 3, while the processing time is compared in Table 4.

It can be seen from Figure 5 that the traditional BD method has no obvious ringing effect, but the image is more blurred, which is the same as with the M−TV method. It can also be seen that the distortion of the image is not improved by the POCS and M−SB methods. In the case of strong turbulence, the method proposed in this paper obviously performs better than the PIV−RR method.

As can be seen from Table 3, the BM values of the M−SB and PIV−RR methods are smaller, while the proposed method has a larger value. The IC value of the proposed method is the largest, while the PIV−RR method has a small value. The GMG values of both PIV−RR and the proposed method are larger than for the other methods. It can be seen from Table 2 that the proposed method also has an obvious advantage in terms of processing time.

As a result, it can be concluded that, from a subjective point of view, the method proposed in this paper performs better than the other methods in terms of image distortion. Objectively speaking, the method proposed in this paper had performed poorly at deblurring, but is stronger in terms of image resolution and sharpness improvement compared to the reconstruction method. In particular, compared to the PIV−RR method, the two methods are comparable in the case of microturbulence. However, under strong turbulence, the method proposed in this paper is obviously better than the PIV−RR method from a subjective point of view. From the perspective of processing speed, the proposed method has an obvious advantage.

### 3.2. Field Tests

**Figure 6:**
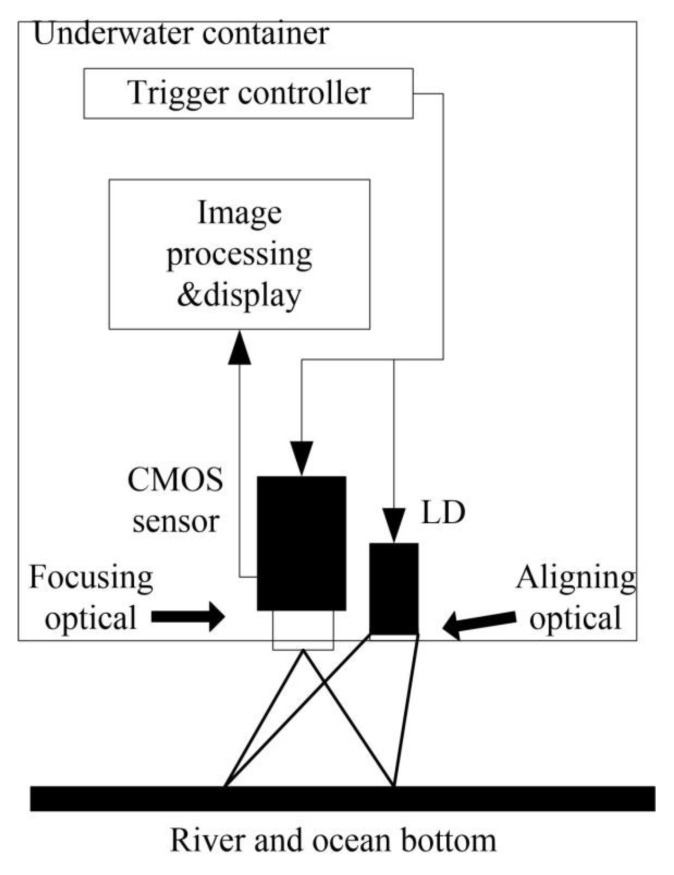
The framework of underwater image detecting system for field tests.

Tests in a turbulent water environment were carried out in the Yangtze River and South China Sea. Sample images were captured by an underwater packaging imaging system. The laser operated at 465−470 nm and CMOS image sensor are enclosed in a waterproof tank, and images captured by the image sensor were transferred to an image processing module. The attenuation coefficient of water is assumed to be a constant that does not change with wavelength in the observation range and can be measured by:

(26) $$K=-\frac{1}{z}In\frac{E(z)}{E(0)},$$

where z is depth; E(z) is irradiance at a depth; and E(0) is irradiance of the surface plane. Figure 6 shows the schematic diagram of the experimental system, with physical properties listed in Table 5. 

**Figure 7:**
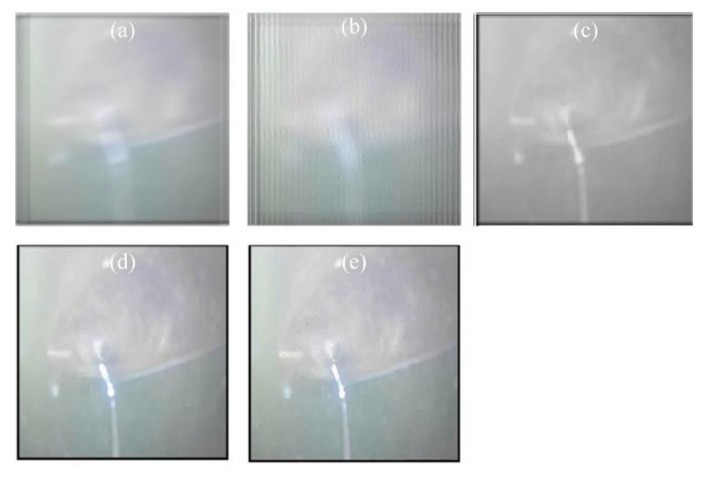
Sample image, restoration and reconstruction results: (**a**) sample image; (**b**) result of M−SB; (**c**) result of M−TV; (**d**) result of PIV−RR; (**e**) result of the proposed method in this paper.

**Figure 8:**
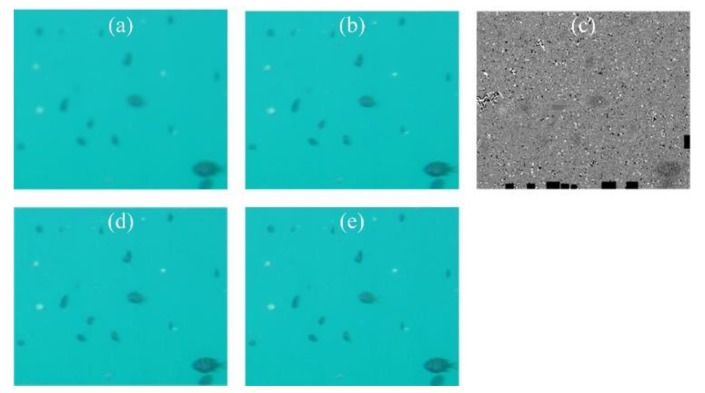
Sample image, restoration and reconstruction results: (**a**) sample image; (**b**) result of M−SB; (**c**) result of M−TV; (**d**) result of PIV−RR; (**e**) result of the proposed method in this paper.

The sample image and processed results are shown in Figure 7 and Figure 8. The evaluation values for the images are listed and compared in Table 6 and Table 7. The processing time of the algorithms are compared in Table 8 and Table 9. After laboratory experiments, the traditional BD and POCS methods are no longer used as comparisons in field experiments.

The experimental results in the river are similar to those in an environment of strong turbulence, while in the ocean the circumstances are more similar to microturbulence, so the effectiveness of laboratory experiments and the robustness of the proposed algorithm can be verified.

In order to further verify the validity of the algorithm, two sets of the TURBID dataset [36] with a turbidity of I_10_ are used for image enhancement and comparison with other latest underwater image enhancement methods. In recent years, the research on underwater image enhancement has mainly focused on mathematical methods such as estimation [37,38,39,40], fusion [41], color correction [42,43,44], and the combination of depth neural network [45,46,47]. In this paper, Accurate Image Super-Resolution Using Very Deep Convolutional Networks (VDSR) [48] is chosen for comparison as a deep neural network method. Zhang et al. [49] proposed a medium transmission estimation method for underwater images based on joint prior distribution, which is also added for comparison. A future research direction is to introduce neural networks and adopt more new datasets, such as the Underwater Image Enhancement Benchmark Dataset (UIEBD) [50,51].

**Figure 9:**
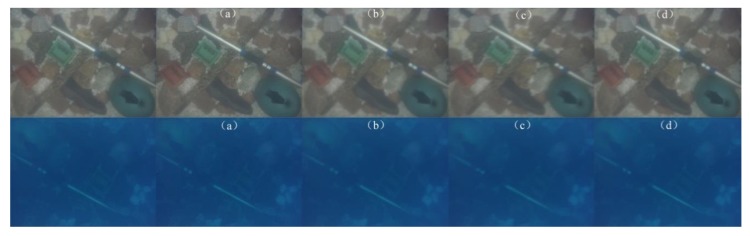
Sample image, restoration and reconstruction results (size 3630 × 2723 pixels) of Chlorophyll (upper row): (**a**) result of VDSR; (**b**) result of Zhang’s method; (**c**) result of PIV−RR; (**d**) result of the proposed method, and that of Deep blue (bottom row): (**a**) result of VDSR; (**b**) result of Zhang’s method; (**c**) result of PIV−RR; (**d**) result of the proposed method.

The processing results are shown in Figure 9 and the evaluation results are given in Table 10. 

It can be seen from the experimental results that the VDSR and PIV methods are inferior, while Zhang’s method and the proposed method show good results, especially for the Chlorophyll dataset. According to the paper that proposed the datasets, the generation of turbidity mainly affects the scattering. Therefore, Zhang’s method for light scattering and the proposed method considering scattering displacement will achieve better recovery effects. The VDSR method, with its uncertain training process, and the PIV method, dependent on measured parameters, cannot achieve good results. It is noted that the time of VDSL is faster than that of the proposed method, but this is after training, and the time of training samples is not included. The method proposed in this paper can be used for both de–distortion and de–blurring, so it is more applicable.

## 4. Conclusions

Combining pixel registration and SVM−KCF algorithms, an underwater turbulence degradation image deformity correction algorithm based on pixel displacement estimation is proposed in this paper. Experimental verification was carried out through a laboratory-simulated turbulent environment and field tests in the river and ocean. Compared with traditional image recovery algorithms, the proposed algorithm can effectively suppress distortion and obtain better objective evaluation index parameters in both micro and strong turbulent environments. The proposed method has an obvious advantage in terms of processing time. Therefore, it can be concluded that the proposed algorithm can effectively suppress the image distortion caused by underwater turbulence, and significantly reduce the processing time, which provides theoretical and technical support for real-time underwater imaging detection.

## Figures and Tables

**Table 1:** Comparison of evaluation results.

Image	Original	BD	POCS	M-SB	M-TV	PIV-RR	Proposed
BM	0.5745	0.3765	0.4910	0.1785	0.2765	0.1643	0.2568
IC	7.1401	8.0018	10.8352	9.3782	11.4782	8.7352	11.2806
GMG	158852	398102	902367	1298701	1176529	1782997	1865205

**Table 2:** Comparison of algorithm running time (min).

Algorithm	Time
BD	11.5
POCS	8.3
M−SB	5.2
M−TV	2.5
PIV−RR	2.9
Proposed	0.9

**Table 3:** Comparison of evaluation results.

Image	Original	BD	POCS	M-SB	M-TV	PIV-RR	Proposed
BM	0.2917	0.1833	0.2783	0.0382	0.1782	0.0922	0.1892
IC	5.5011	3.0283	6.3872	8.9862	7.1382	4.9012	9.0023
GMG	55996	47893	897438	789306	903754	1078322	9799371

**Table 4:** Comparison of algorithm running time (min).

Algorithm	Time
BD	5.3
POCS	4.8
M−SB	5.2
M−TV	1.9
PIV−RR	2.3
Proposed	1.1

**Table 5:** Physical properties of experimental system.

Parameters	Value
Water attenuation (t)	2.9 m^−1^
LD power ($P_{0}$)	1 W
Operating Voltage (V)	12 V
Angle of viewing (θ)	90°
Distance between LD and CCD ($d_{{}_{0}}$)	1 cm

**Table 6:** Comparison of evaluation results.

Image	Original	M-SB	M-TV	PIV-RR	Proposed
BM	0.8032	0.3891	0.5710	0.2835	0.3978
IC	4.8923	12.9011	9.7893	8.3081	15.8923
GMG	28934	974033	440219	1098364	1063774

**Table 7:** Comparison of evaluation results.

Image	Original	M-SB	M-TV	PIV-RR	Proposed
BM	0.4903	0.0671	1.9032	0.0212	0.0298
IC	2.9903	11.2341	4.2088	7.0023	18.9032
GMG	57891	520301	309872	908891	943374

**Table 8:** Comparison of algorithm running time (min).

Algorithm	Time
M−SB	4.5
M−TV	2.4
PIV−RR	3.7
Proposed	0.5

**Table 9:** Comparison of algorithm running time (min).

Algorithm	Time
M−SB	6.3
M−TV	1.5
PIV−RR	2.5
Proposed	1.3

**Table 10:** Evaluation results and running time (min).

		VDSR	Zhang	PIV−RR	Proposed
Deep blue	BM	0.8539	0.3748	0.6847	0.3184
	IC	5.2342	9.3435	6.2343	9.2344
	Time	0.5	1.3	2.1	0.9
Chlorophyll	BM	0.8374	0.1342	0.5890	0.1193
	IC	3.9877	5.2342	6.2342	11.3454
	Time	0.3	2.5	1.8	0.5
